# [(1,2,5,6-η)-Cyclo­octa-1,5-diene](1-ethyl-4-isobutyl-1,2,4-triazol-5-yl­idene)(tri­phenyl­phosphane)rhodium(I) tetra­fluorido­borate

**DOI:** 10.1107/S2414314624007454

**Published:** 2024-08-02

**Authors:** Timothy G. Lerch, Michael Gau, Daniel R. Albert, Edward Rajaseelan

**Affiliations:** a Department of Chemistry Millersville University,Millersville PA 17551 USA; b Department of Chemistry, University of Pennsylvania, Philadelphia, PA 19104, USA; Sunway University, Malaysia

**Keywords:** crystal structure, rhodium, *N*-heterocyclic carbenes, cationic complexes

## Abstract

A new, cationic *N*-heterocyclic carbene Rh^I^ complex with a tetra­fluorido­borate counter-anion, [Rh(C_8_H_12_)(C_8_H_15_N_3_)(C_18_H_15_P)]BF_4_, has been synthesized and structurally characterized. There are two independent ion pairs in the asymmetric unit. Each complex cation exhibits a distorted square-planar conformation around the Rh^I^ atom. Bond lengths and bond angles are as expected for an Rh–NHC complex. There are several close, non-standard C—H⋯F hydrogen-bonding inter­actions between the ions. One of the tetra­fluorido­borate anions shows statistical disorder of the F atoms.

## Abstract 2

There are two independent ion pairs in the asymmetric unit with each complex cation exhibiting a distorted square-planar conformation around the rhodium(I) atom.

## Graphical abstract

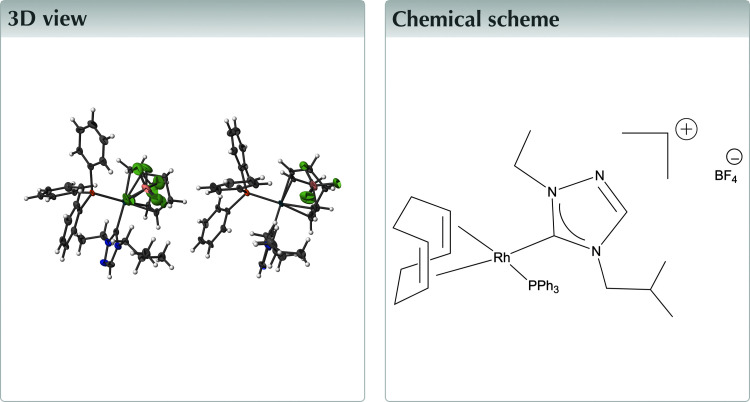

## Structure description

*N*-heterocyclic carbene (NHC) complexes of transition metals have been studied extensively in homogeneous catalysis, especially in transfer hydrogenation of unsaturated bonds (Cazin, 2013 ▸; Diez-González *et al.*, 2009 ▸; Rovis & Nolan, 2013 ▸; Ruff *et al.*, 2016 ▸; Zuo *et al.*, 2014 ▸). Their catalytic activity in the transfer hydrogenation of ketones and imines has also been studied and reported (Albrecht *et al.*, 2002 ▸; Gnanamgari *et al.*, 2007 ▸). The NHC ligands can be tuned both sterically and electronically by having different substituents on the nitro­gen atoms (Diez-González & Nolan, 2007 ▸; Gusev, 2009 ▸). Many imidazole- and triazole-based NHC rhodium and iridium complexes have been synthesized and structurally characterized (Chianese *et al.*, 2004 ▸; Herrmann *et al.*, 2006 ▸; Wang & Lin 1998 ▸). We continue to synthesize new imidazole- and triazole-based NHC complexes of rhodium and iridium, to study the effect of different substituents on the NHCs and the other ligands coordinating to the metal in transfer hydrogenation reactions (Nichol *et al.*, 2009 ▸, 2010 ▸, 2011 ▸, 2012 ▸; Idrees *et al.*, 2017*a* ▸,*b* ▸; Rood *et al.*, 2021 ▸; Rushlow *et al.*, 2021 ▸; Newman *et al.*, 2021 ▸; Castaldi *et al.*, 2021 ▸; Maynard *et al.*, 2023 ▸; Lerch *et al.*, 2024*a* ▸,*b* ▸).

**Figure 1:**
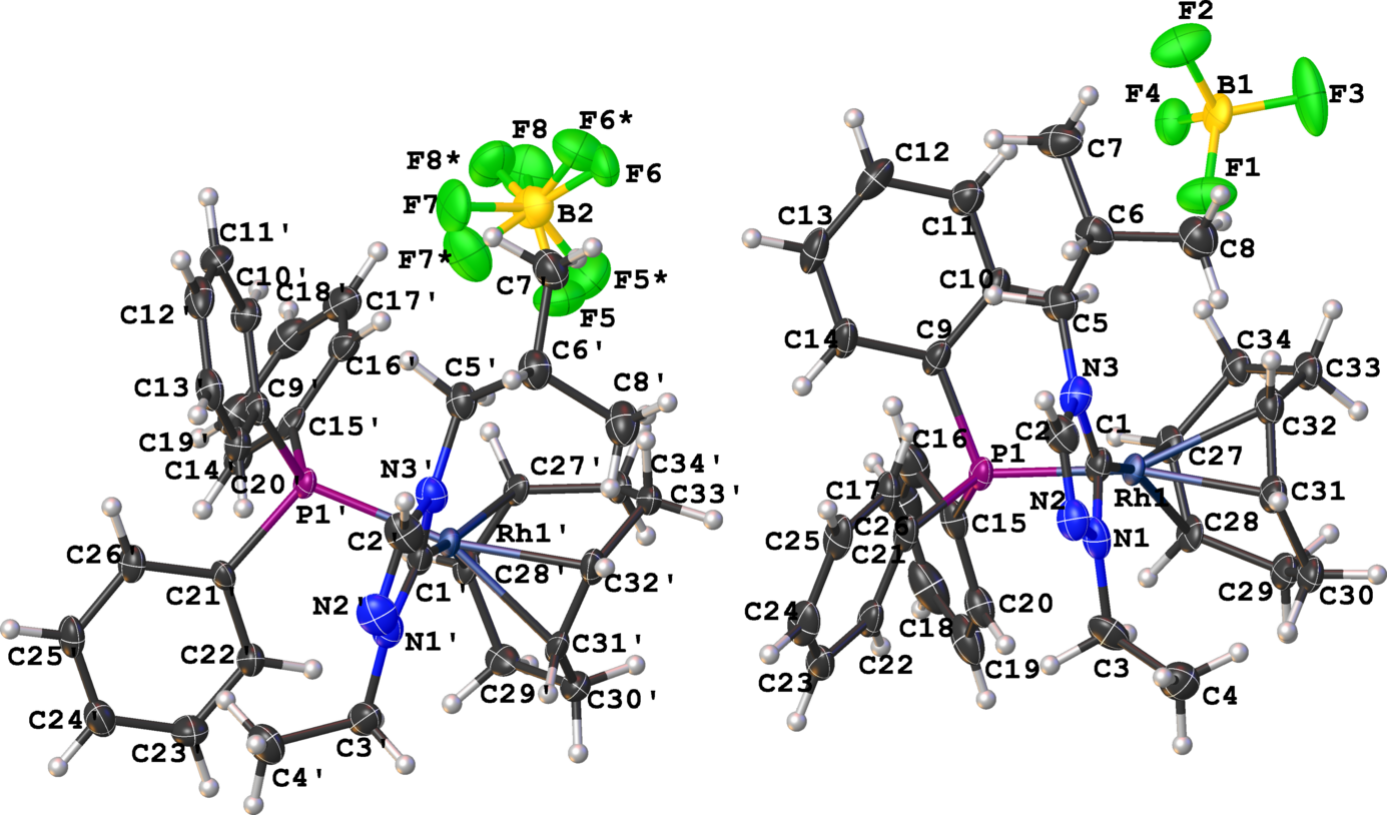
Mol­ecular structures of the four ions comprising the asymmetric unit of the title salt showing the atom-labeling scheme. Cation *A*, containing Rh1, is shown on the right. Displacement ellipsoids are drawn at the 50% probability level. Both orientations of the statistically disordered B2-tetra­fluorido­borate anion are shown.

The title complex, [Rh(C_8_H_12_)(C_8_H_15_N_3_)(C_18_H_15_P)]BF_4_ (**2**), comprises a cationic Rh^I^ complex and a tetra­fluorido­borate counter-anion, with mol­ecular structures illustrated in Fig. 1 ▸; there are two independent ion pairs in the asymmetric unit. The coordination sphere around the Rh^I^ cation, formed by the bidentate COD, NHC, and tri­phenyl­phosphane ligands, results in a distorted square-planar conformation, characterized by C_NHC_—Rh—P bond angles of 89.6 (2)° for cation *A* and 89.8 (2)° for cation *B.* The N—C—N bond angles of the NHC ligand are 102.4 (7) and 103.8 (6)° for cations *A* and *B*, respectively. Other selected bond lengths in cations *A* and *B* are Rh—C_NHC_ = 2.030 (8) and 2.043 (8) Å, Rh—P = 2.3211 (18) and 2.3260 (15) Å, respectively.

**Figure 2:**
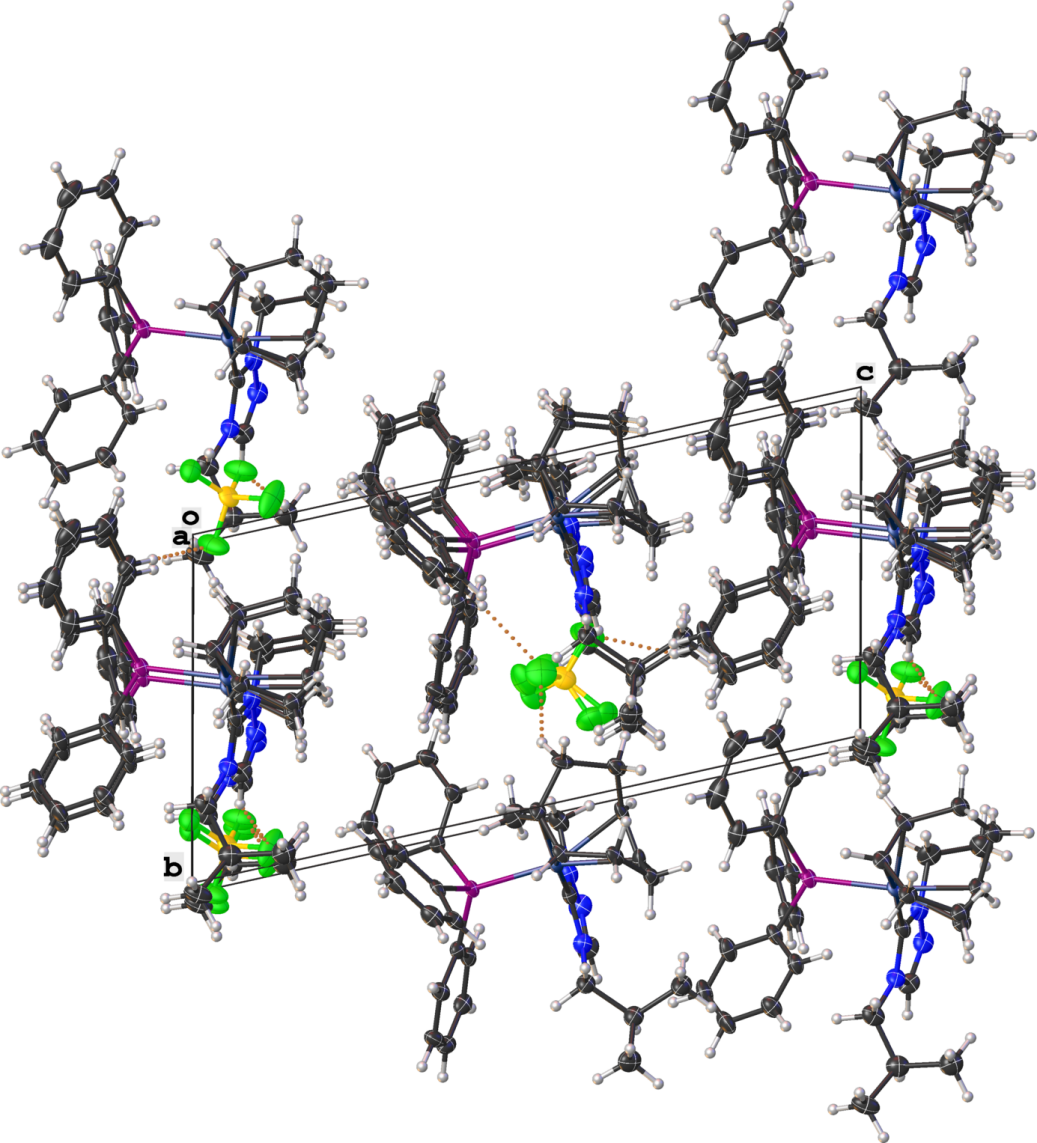
Mol­ecular packing diagram of the title salt visualized along the *a* axis with non-standard hydrogen-bonding inter­actions shown as dotted orange lines.

Fig. 2 ▸ shows a mol­ecular packing diagram of the salt viewed along the *a* axis, with several close C—H⋯F contacts (likely, non-standard hydrogen-bonding inter­actions) stabilizing the orientation of the [BF_4_]^−^ anions with respect to the Rh^I^ complex cations. The non-standard hydrogen-bonding inter­actions are shown as dotted orange lines in Fig. 2 ▸ and are summarized in Table 1 ▸.

**Figure 3:**
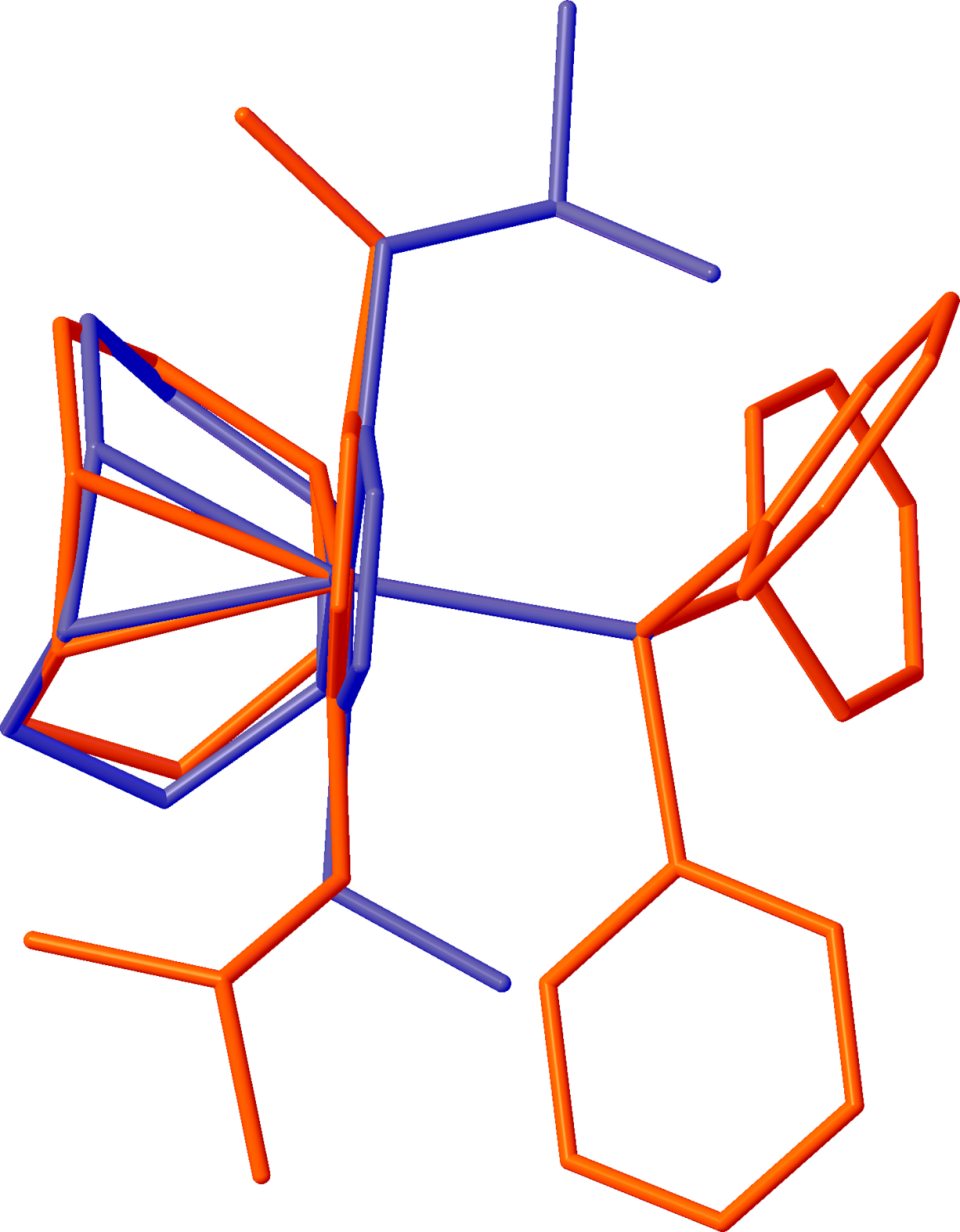
Overlay of complex *A* of the salt, shown in red, with previously characterized (Lerch *et al.*, 2024*c* ▸) neutral complex (**1**), showing that the Rh—C_NHC_ bond has rotated 180° in the synthesis of the cationic complex from the neutral precursor. The overlay is constructed with C_NHC_, Rh, and Cl/P atoms matched between the neutral complex (**1**) and complex *A* of the title salt (**2**) with an r.m.s. deviation of 0.029 Å between the matched atoms.

**Figure 4:**
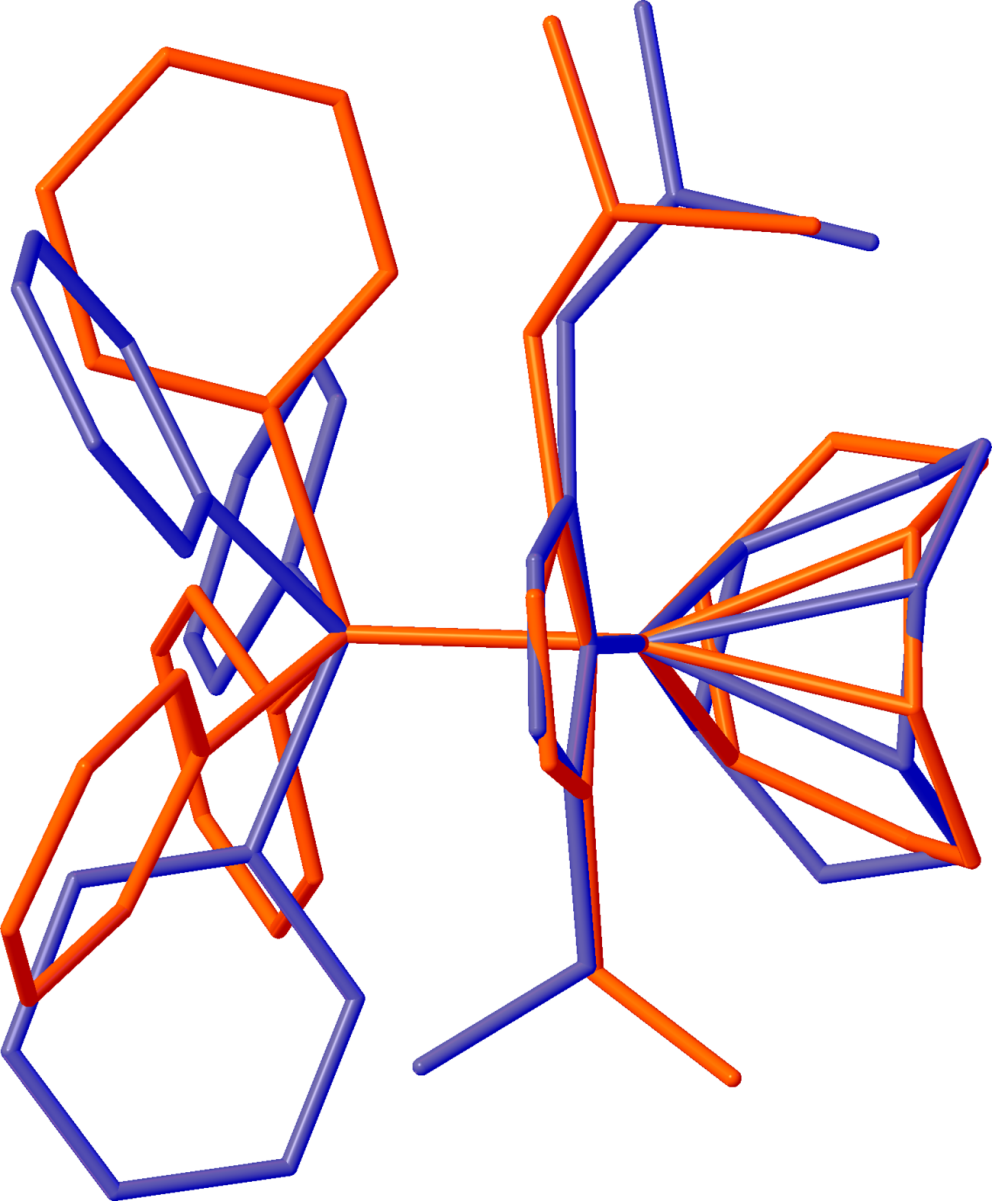
Overlay of complex *A*, shown in red, with complex *B* of the title salt showing the difference in orientation of the ethyl and isobutyl substituents with respect to the NHC ligand. The overlay is constructed with C_NHC_, Rh, and P atoms matched between the two cations of with an r.m.s. deviation of 0.008 Å between the matched atoms.

An overlay of the neutral precursor complex, chlorido­[(1,2,5,6-η)-cyclo­octa-1,5-diene](1-ethyl-4-isobutyl-1,2,4-tri­azol-5-yl­idene)rhodium(I) (**1**) (Lerch *et al.*, 2024*c* ▸), and cation *A* of the title salt (**2**) (Fig. 3 ▸), shows that in (**2**) the Rh—C_NHC_ bond, and hence the entire NHC ligand, is rotated by almost 180° with respect to the remainder of the complex as visualized by the ethyl and isobutyl substituents not overlapping when the C_NHC_, Rh, and Cl/P atoms are matched with the neutral complex (**1**). A similar rotation is observed in complex *B*. The two cations in the asymmetric-unit exhibit different configurations of the ethyl and isobutyl substituents with cation *A* showing a *syn*-configuration and cation *B* showing an *anti*-configuration with respect to each other when the C_NHC_, Rh, and P atoms are matched between *A* and *B*, as seen in Fig. 4 ▸.

## Synthesis and crystallization

**Figure 5:**
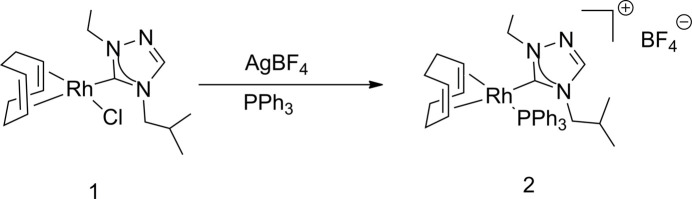
Reaction scheme for the synthesis of the title salt (**2**).

The synthesis and structure of chlorido­[(1,2,5,6-η)-cycloocta-1,5-diene](1-ethyl-4-isobutyl-1,2,4-triazol-5-yl­idene)rhodium(I) (**1**) have been published (Lerch *et al.* 2024*c* ▸). All other compounds used in the syntheses, as shown in Fig. 5 ▸, were obtained from Sigma-Aldrich and Strem and used as received; all syntheses were performed under a nitro­gen atmosphere. NMR spectra were recorded at room temperature in CDCl_3_ on a 400 MHz (operating at 100 MHz for ^1^H and ^13^C, and 162 MHz for ^31^P) Varian spectrometer and referenced to the residual solvent peak (δ in p.p.m.). The title salt (**2**) was crystallized by slow diffusion of pentane into a CH_2_Cl­_2_ solution.

[(1,2,5,6-η)-Cyclo­octa-1,5-diene](1-ethyl-4-isobutyl-1,2,4-tri­azol-5-yl­idene)(tri­phenyl­phosphane)rhodium(I) tetra­fluorido­borate (**2**): Tri­phenyl­phosphane (0.097 g, 0.372 mmol) and AgBF_4_ (0.072 g, 0.372 mmol) were added to (**1**) (0.149 g, 0.372 mmol) in CH_2_Cl_2_ (15 ml). The solution was stirred in the dark for 1.5 h. The resulting mixture was filtered through Celite and the solvent was removed under reduced pressure. The bright-yellow solid product (**2**) was dried under vacuum. Yield: 0.266 g (100%). ^1^H NMR: δ 8.08 (*s*, 1H, N—C3H—N), 7.48–7.25 (*m*, 15H, H_aromatic_), 4.72 (*q*, 2H, N—CH_2_ of eth­yl), 4.47 (*d*, 2H, N—CH_2_ of isobut­yl), 4.24 (*m*, 2H, CH of COD), 4.08 (*m*, 2H, CH of COD), 2.60 (*m*, 4H, CH_2_ of COD), 2.48 (*m*, 2H, CH_2_ of COD), 2.23 (*m*, 2H, CH_2_ of COD), 2.06 (*m*, 1H, CH of isobut­yl), 1.23 (*t*, 3H, CH_3_ of eth­yl), 0.92 (*d*, 6H, CH_3_ of isobut­yl). ^13^C NMR: δ 181.43 (*d*, Rh—C, *J*_C—Rh_ = 49.3 Hz), 143.88 (N—C3H—N), 133.47–128.49 (C_aromatic_), 95.54, 95.46, 95.18, 95.06 (CH of COD), 55.70 (N—CH_2_ of isobut­yl), 48.11 (N—CH_2_ of eth­yl), 30.85, 30.50, 30.48, 30.19 (CH_2_ of COD), 29.22 (CH of isobut­yl), 19.98 (CH_3_ of isobut­yl), 13.93 (CH_3_ of eth­yl).^31^P NMR: δ 25.10 (*d*, *J*_Rh—P_ = 152.77 Hz).

## Refinement

Refinement details are summarized in Table 2 ▸. One of the tetra­fluorido­borate anions shows positional disorder of the F atoms over adjacent sites in a 1:1 ratio. The absolute structure was determined based on differences in Friedel pairs included in the data set (Parsons *et al.*, 2013 ▸). The maximum and minimum electron density peaks of 2.51 and 1.08 e Å^−3^, respectively, were each located 0.87 Å from the Rh1 atom. Two reflections, *i.e*. 0
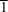
1 and 1
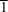
0, were omitted from the final cycles of refinement owing to poor agreement.

## Supplementary Material

Crystal structure: contains datablock(s) I. DOI: 10.1107/S2414314624007454/tk4108sup1.cif

Structure factors: contains datablock(s) I. DOI: 10.1107/S2414314624007454/tk4108Isup2.hkl

CCDC reference: 2374311

Additional supporting information:  crystallographic information; 3D view; checkCIF report

## Figures and Tables

**Table 1:** Hydrogen-bond geometry (Å, °)

*D*—H⋯*A*	*D*—H	H⋯*A*	*D*⋯*A*	*D*—H⋯*A*
C2—H2⋯F3^i^	0.95	2.36	3.177 (8)	144
C8—H8*C*⋯F1	0.98	2.50	3.404 (10)	153
C13—H13⋯F5*	0.95	2.52	3.396 (17)	153
C20—H20⋯F2^ii^	0.95	2.34	3.291 (9)	178
C16′—H16′⋯F7*	0.95	2.40	3.351 (17)	177
C29′—H29*D*⋯F8^ii^	0.99	2.51	3.37 (2)	146

Symmetry codes: (i) 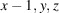; (ii) 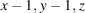.

**Table 2:** Experimental details

Crystal data	
Chemical formula	[Rh(C_8_H_12_)(C_8_H_15_N_3_)(C_18_H_15_P)]BF_4_
*M*_r_	713.39
Crystal system, space group	Triclinic, *P*1
Temperature (K)	100
*a*, *b*, *c* (Å)	10.0296 (3), 10.3718 (3), 18.1424 (4)
α, β, γ (°)	99.270 (2), 93.906 (2), 118.766 (3)
*V* (Å^3^)	1609.47 (9)
*Z*	2
Radiation type	Mo *K*α
μ (mm^−1^)	0.63
Crystal size (mm)	0.23 × 0.13 × 0.12

Data collection	
Diffractometer	Rigaku XtaLAB Synergy-S
Absorption correction	Multi-scan (*CrysAlis PRO*; Rigaku OD, 2024 ▸)
*T*_min_, *T*_max_	0.823, 1.000
No. of measured, independent and observed [*I* > 2σ(*I*)] reflections	46659, 15088, 13740
*R*_int_	0.047
(sin θ/λ)_max_ (Å^−1^)	0.667

Refinement	
*R*[*F*^2^ > 2σ(*F*^2^)], *wR*(*F*^2^), *S*	0.049, 0.124, 1.03
No. of reflections	15088
No. of parameters	835
No. of restraints	135
H-atom treatment	H-atom parameters constrained
Δρ_max_, Δρ_min_ (e Å^−3^)	2.51, −1.08
Absolute structure	Flack *x* determined using 5836 quotients [(*I*^+^)−(*I*^−^)]/[(*I*^+^)+(*I*^−^)] (Parsons *et al.*, 2013 ▸)
Absolute structure parameter	−0.029 (12)

Computer programs: *CrysAlis PRO* (Rigaku OD, 2024 ▸), *SHELXT* (Sheldrick, 2015*a* ▸), *SHELXL2018/3* (Sheldrick, 2015*b* ▸), *OLEX2* (Dolomanov *et al.*, 2009 ▸) and *publCIF* (Westrip, 2010 ▸).

## full crystallographic data

### [(1,2,5,6-η)-Cycloocta-1,5-diene](1-ethyl-4-isobutyl-1,2,4-triazol-5-ylidene)(triphenylphosphane)rhodium(I) tetrafluoridoborate Crystal data

**Table:**

[Rh(C_8_H_12_)(C_8_H_15_N_3_)(C_18_H_15_P)]BF_4_	*Z* = 2
*M**_r_* = 713.39	*F*(000) = 736
Triclinic, *P*1	*D*_x_ = 1.472 Mg m^−^^3^
*a* = 10.0296 (3) Å	Mo *K*α radiation, λ = 0.71073 Å
*b* = 10.3718 (3) Å	Cell parameters from 24773 reflections
*c* = 18.1424 (4) Å	θ = 2.2–28.3°
α = 99.270 (2)°	µ = 0.63 mm^−^^1^
β = 93.906 (2)°	*T* = 100 K
γ = 118.766 (3)°	Block, yellow
*V* = 1609.47 (9) Å^3^	0.23 × 0.13 × 0.12 mm

### [(1,2,5,6-η)-Cycloocta-1,5-diene](1-ethyl-4-isobutyl-1,2,4-triazol-5-ylidene)(triphenylphosphane)rhodium(I) tetrafluoridoborate Data collection

**Table:**

Rigaku XtaLAB Synergy-S diffractometer	13740 reflections with *I* > 2σ(*I*)
Detector resolution: 10.0 pixels mm^-1^	*R*_int_ = 0.047
ω scans	θ_max_ = 28.3°, θ_min_ = 2.3°
Absorption correction: multi-scan (CrysAlisPro; Rigaku OD, 2024)	*h* = −13→13
*T*_min_ = 0.823, *T*_max_ = 1.000	*k* = −13→13
46659 measured reflections	*l* = −24→24
15088 independent reflections	

### [(1,2,5,6-η)-Cycloocta-1,5-diene](1-ethyl-4-isobutyl-1,2,4-triazol-5-ylidene)(triphenylphosphane)rhodium(I) tetrafluoridoborate Refinement

**Table:**

Refinement on *F*^2^	Hydrogen site location: inferred from neighbouring sites
Least-squares matrix: full	H-atom parameters constrained
*R*[*F*^2^ > 2σ(*F*^2^)] = 0.049	*w* = 1/[σ^2^(*F*_o_^2^) + (0.0858*P*)^2^ + 0.5161*P*] where *P* = (*F*_o_^2^ + 2*F*_c_^2^)/3
*wR*(*F*^2^) = 0.124	(Δ/σ)_max_ = 0.001
*S* = 1.03	Δρ_max_ = 2.51 e Å^−^^3^
15088 reflections	Δρ_min_ = −1.08 e Å^−^^3^
835 parameters	Absolute structure: Flack *x* determined using 5836 quotients [(*I*^+^)-(*I*^-^)]/[(*I*^+^)+(*I*^-^)] (Parsons *et al.*, 2013)
135 restraints	Absolute structure parameter: −0.029 (12)

### [(1,2,5,6-η)-Cycloocta-1,5-diene](1-ethyl-4-isobutyl-1,2,4-triazol-5-ylidene)(triphenylphosphane)rhodium(I) tetrafluoridoborate Special details

**Table:**

Geometry. All esds (except the esd in the dihedral angle between two l.s. planes) are estimated using the full covariance matrix. The cell esds are taken into account individually in the estimation of esds in distances, angles and torsion angles; correlations between esds in cell parameters are only used when they are defined by crystal symmetry. An approximate (isotropic) treatment of cell esds is used for estimating esds involving l.s. planes.

### [(1,2,5,6-η)-Cycloocta-1,5-diene](1-ethyl-4-isobutyl-1,2,4-triazol-5-ylidene)(triphenylphosphane)rhodium(I) tetrafluoridoborate Fractional atomic coordinates and isotropic or equivalent isotropic displacement parameters (Å^2^)

**Table:**

	*x*	*y*	*z*	*U*_iso_*/*U*_eq_	Occ. (<1)
Rh1	0.37193 (4)	0.44291 (4)	1.05195 (2)	0.01839 (12)	
P1	0.2676 (2)	0.3571 (2)	0.92393 (10)	0.0209 (4)	
N1	0.1208 (7)	0.5098 (7)	1.0896 (3)	0.0287 (12)	
N2	0.0659 (7)	0.6073 (7)	1.0954 (3)	0.0297 (12)	
N3	0.2903 (8)	0.6987 (7)	1.0545 (3)	0.0276 (12)	
C1	0.2574 (9)	0.5596 (9)	1.0644 (4)	0.0250 (16)	
C2	0.1698 (7)	0.7192 (8)	1.0741 (4)	0.0281 (14)	
H2	0.165092	0.807911	1.071937	0.034*	
C3	0.0256 (8)	0.3582 (8)	1.1035 (5)	0.0345 (16)	
H3A	0.083615	0.303285	1.098427	0.041*	
H3B	−0.069026	0.301672	1.064678	0.041*	
C4	−0.0190 (9)	0.3642 (9)	1.1814 (5)	0.0381 (17)	
H4A	−0.088158	0.261541	1.187122	0.057*	
H4B	−0.071816	0.422936	1.187507	0.057*	
H4C	0.073962	0.412030	1.219987	0.057*	
C5	0.4182 (8)	0.7952 (9)	1.0209 (4)	0.0312 (15)	
H5A	0.381174	0.781082	0.966642	0.037*	
H5B	0.496867	0.763687	1.023905	0.037*	
C6	0.4922 (8)	0.9587 (8)	1.0579 (4)	0.0327 (15)	
H6	0.410132	0.987045	1.060647	0.039*	
C7	0.6059 (9)	1.0527 (9)	1.0102 (5)	0.0396 (18)	
H7A	0.678330	1.016096	1.000976	0.059*	
H7B	0.663184	1.158704	1.037307	0.059*	
H7C	0.549224	1.043543	0.961654	0.059*	
C8	0.5722 (9)	0.9875 (10)	1.1382 (5)	0.0399 (17)	
H8A	0.497170	0.924182	1.167018	0.060*	
H8B	0.615963	1.093976	1.162930	0.060*	
H8C	0.655018	0.962959	1.136197	0.060*	
C9	0.3857 (8)	0.4998 (8)	0.8729 (4)	0.0251 (13)	
C10	0.5391 (8)	0.6020 (8)	0.9045 (4)	0.0259 (13)	
H10	0.580519	0.595211	0.951506	0.031*	
C11	0.6322 (8)	0.7130 (8)	0.8689 (4)	0.0316 (15)	
H11	0.737716	0.779861	0.890352	0.038*	
C12	0.5703 (10)	0.7264 (9)	0.8008 (4)	0.0371 (17)	
H12	0.632014	0.805371	0.777058	0.045*	
C13	0.4172 (9)	0.6229 (10)	0.7684 (4)	0.0351 (16)	
H13	0.375949	0.629427	0.721327	0.042*	
C14	0.3259 (8)	0.5123 (9)	0.8033 (4)	0.0313 (15)	
H14	0.221501	0.443304	0.780758	0.038*	
C15	0.2629 (7)	0.1842 (8)	0.8756 (4)	0.0262 (13)	
C16	0.3457 (9)	0.1843 (10)	0.8164 (4)	0.0366 (17)	
H16	0.398519	0.272464	0.797102	0.044*	
C17	0.3504 (9)	0.0567 (11)	0.7866 (5)	0.047 (2)	
H17	0.407399	0.057897	0.746747	0.057*	
C18	0.2734 (10)	−0.0747 (10)	0.8133 (5)	0.047 (2)	
H18	0.278777	−0.161938	0.792593	0.056*	
C19	0.1903 (10)	−0.0755 (10)	0.8698 (5)	0.0401 (19)	
H19	0.136714	−0.164718	0.888261	0.048*	
C20	0.1825 (8)	0.0532 (8)	0.9013 (4)	0.0278 (14)	
H20	0.122405	0.050244	0.939923	0.033*	
C21	0.0720 (8)	0.3266 (8)	0.8986 (4)	0.0243 (14)	
C22	−0.0583 (8)	0.1847 (8)	0.8783 (4)	0.0271 (14)	
H22	−0.048015	0.097459	0.873023	0.033*	
C23	−0.2036 (8)	0.1692 (9)	0.8656 (4)	0.0327 (16)	
H23	−0.292064	0.071238	0.852093	0.039*	
C24	−0.2216 (8)	0.2935 (10)	0.8723 (4)	0.0342 (16)	
H24	−0.321888	0.281275	0.863550	0.041*	
C25	−0.0923 (8)	0.4376 (10)	0.8920 (4)	0.0332 (16)	
H25	−0.103899	0.524097	0.897197	0.040*	
C26	0.0545 (8)	0.4537 (8)	0.9039 (4)	0.0273 (14)	
H26	0.143124	0.551656	0.915819	0.033*	
C27	0.5324 (10)	0.3584 (9)	1.0335 (5)	0.0249 (9)	
H27	0.526628	0.315767	0.979107	0.030*	
C28	0.4182 (8)	0.2608 (8)	1.0673 (4)	0.0248 (8)	
H28	0.344994	0.161411	1.032437	0.030*	
C29	0.4340 (8)	0.2524 (8)	1.1503 (4)	0.0290 (9)	
H29A	0.371626	0.145953	1.154031	0.035*	
H29B	0.543194	0.286840	1.169527	0.035*	
C30	0.3829 (8)	0.3469 (8)	1.2002 (4)	0.0292 (9)	
H30A	0.433736	0.370508	1.253181	0.035*	
H30B	0.270166	0.286561	1.198645	0.035*	
C31	0.4201 (9)	0.4951 (9)	1.1771 (4)	0.0283 (9)	
H31	0.368593	0.547936	1.202330	0.034*	
C32	0.5541 (8)	0.5877 (8)	1.1532 (4)	0.0286 (9)	
H32	0.581468	0.695999	1.163289	0.034*	
C33	0.6937 (8)	0.5643 (8)	1.1506 (4)	0.0280 (9)	
H33A	0.695674	0.507604	1.189269	0.034*	
H33B	0.789246	0.663942	1.163880	0.034*	
C34	0.6924 (8)	0.4783 (8)	1.0726 (4)	0.0267 (8)	
H34A	0.739640	0.550969	1.040035	0.032*	
H34B	0.756311	0.430809	1.079328	0.032*	
Rh1'	−0.30754 (4)	0.16819 (4)	0.54548 (2)	0.01794 (12)	
P1'	−0.33122 (17)	0.18483 (19)	0.41940 (9)	0.0182 (3)	
N1'	−0.5965 (7)	0.1941 (7)	0.5643 (3)	0.0277 (12)	
N2'	−0.6483 (7)	0.2947 (8)	0.5817 (4)	0.0350 (14)	
N3'	−0.3968 (6)	0.4103 (7)	0.5856 (3)	0.0263 (11)	
C1'	−0.4451 (8)	0.2603 (9)	0.5656 (4)	0.0231 (15)	
C2'	−0.5245 (8)	0.4232 (9)	0.5937 (4)	0.0323 (15)	
H2'	−0.521925	0.516955	0.606750	0.039*	
C3'	−0.7094 (8)	0.0336 (9)	0.5485 (4)	0.0318 (15)	
H3'A	−0.755330	0.008215	0.594342	0.038*	
H3'B	−0.656619	−0.024956	0.536251	0.038*	
C4'	−0.8378 (9)	−0.0117 (10)	0.4826 (5)	0.0388 (17)	
H4'A	−0.911323	−0.120026	0.474029	0.058*	
H4'B	−0.793067	0.010723	0.436718	0.058*	
H4'C	−0.891248	0.044967	0.494734	0.058*	
C5'	−0.2378 (8)	0.5324 (8)	0.5908 (4)	0.0270 (13)	
H5'A	−0.230872	0.577080	0.545979	0.032*	
H5'B	−0.168451	0.489484	0.589641	0.032*	
C6'	−0.1816 (8)	0.6576 (8)	0.6627 (4)	0.0298 (14)	
H6'	−0.257116	0.694491	0.665291	0.036*	
C7'	−0.0273 (9)	0.7875 (9)	0.6568 (5)	0.0385 (17)	
H7'A	0.050135	0.755519	0.657473	0.058*	
H7'B	0.003866	0.872260	0.699807	0.058*	
H7'C	−0.035695	0.818915	0.609407	0.058*	
C8'	−0.1733 (10)	0.5997 (9)	0.7340 (4)	0.0368 (17)	
H8'A	−0.275564	0.517405	0.736632	0.055*	
H8'B	−0.138914	0.681962	0.778749	0.055*	
H8'C	−0.099948	0.562693	0.732563	0.055*	
C9'	−0.3310 (7)	0.3517 (7)	0.3987 (3)	0.0223 (12)	
C10'	−0.2087 (8)	0.4634 (7)	0.3730 (4)	0.0259 (13)	
H10'	−0.124489	0.450046	0.361608	0.031*	
C11'	−0.2089 (8)	0.5946 (8)	0.3640 (4)	0.0305 (15)	
H11'	−0.125718	0.669254	0.345831	0.037*	
C12'	−0.3316 (9)	0.6160 (8)	0.3818 (4)	0.0324 (15)	
H12'	−0.330661	0.706217	0.376793	0.039*	
C13'	−0.4533 (8)	0.5063 (8)	0.4064 (4)	0.0283 (14)	
H13'	−0.536914	0.520409	0.418097	0.034*	
C14'	−0.4544 (8)	0.3744 (8)	0.4143 (4)	0.0243 (13)	
H14'	−0.540121	0.298502	0.430423	0.029*	
C15'	−0.1893 (8)	0.1649 (7)	0.3684 (3)	0.0224 (12)	
C16'	−0.0320 (8)	0.2722 (8)	0.3934 (4)	0.0276 (14)	
H16'	−0.002065	0.358709	0.432365	0.033*	
C17'	0.0795 (8)	0.2505 (9)	0.3605 (5)	0.0348 (16)	
H17'	0.185545	0.322988	0.377386	0.042*	
C18'	0.0380 (9)	0.1254 (10)	0.3039 (5)	0.0378 (17)	
H18'	0.115320	0.111868	0.282248	0.045*	
C19'	−0.1165 (9)	0.0195 (9)	0.2786 (4)	0.0324 (15)	
H19'	−0.145555	−0.066225	0.239251	0.039*	
C20'	−0.2290 (8)	0.0395 (8)	0.3113 (4)	0.0263 (13)	
H20'	−0.334744	−0.034021	0.294311	0.032*	
C21'	−0.5153 (6)	0.0283 (7)	0.3674 (3)	0.0194 (11)	
C22'	−0.5830 (7)	−0.1071 (8)	0.3910 (4)	0.0270 (13)	
H22'	−0.533669	−0.116060	0.434959	0.032*	
C23'	−0.7211 (8)	−0.2292 (8)	0.3516 (4)	0.0336 (16)	
H23'	−0.764379	−0.321992	0.367477	0.040*	
C24'	−0.7962 (8)	−0.2154 (9)	0.2886 (4)	0.0320 (15)	
H24'	−0.891240	−0.298811	0.261693	0.038*	
C25'	−0.7340 (8)	−0.0826 (8)	0.2652 (4)	0.0292 (14)	
H25'	−0.786931	−0.074098	0.222446	0.035*	
C26'	−0.5930 (8)	0.0413 (8)	0.3036 (4)	0.0249 (13)	
H26'	−0.550025	0.133120	0.286803	0.030*	
C27'	−0.0969 (7)	0.1462 (7)	0.5524 (4)	0.0212 (12)	
H27'	−0.026273	0.196341	0.517012	0.025*	
C28'	−0.2235 (9)	0.0073 (8)	0.5178 (4)	0.0238 (15)	
H28'	−0.226223	−0.024195	0.462387	0.029*	
C29'	−0.3087 (9)	−0.1220 (8)	0.5551 (4)	0.0287 (15)	
H29C	−0.251029	−0.176795	0.557268	0.034*	
H29D	−0.411092	−0.192822	0.523498	0.034*	
C30'	−0.3314 (8)	−0.0732 (8)	0.6355 (4)	0.0285 (14)	
H30C	−0.421029	−0.159097	0.648284	0.034*	
H30D	−0.239049	−0.046110	0.671854	0.034*	
C31'	−0.3579 (7)	0.0599 (8)	0.6437 (3)	0.0255 (13)	
H31'	−0.460695	0.036623	0.657588	0.031*	
C32'	−0.2456 (9)	0.2096 (9)	0.6699 (4)	0.0263 (16)	
H32'	−0.283070	0.272386	0.699277	0.032*	
C33'	−0.0763 (8)	0.2717 (8)	0.6884 (4)	0.0236 (13)	
H33C	−0.050708	0.264014	0.740640	0.028*	
H33D	−0.022521	0.379961	0.687040	0.028*	
C34'	−0.0169 (7)	0.1899 (8)	0.6338 (4)	0.0251 (13)	
H34C	0.095348	0.256144	0.636344	0.030*	
H34D	−0.032710	0.097768	0.650280	0.030*	
F1	0.7916 (6)	0.8417 (6)	1.0710 (4)	0.0515 (13)	
F2	0.9662 (7)	1.0421 (6)	1.0313 (4)	0.0536 (14)	
F3	1.0502 (8)	0.9503 (8)	1.1177 (3)	0.071 (2)	
F4	0.9502 (5)	0.8151 (5)	0.9960 (3)	0.0373 (10)	
B1	0.9416 (10)	0.9128 (10)	1.0536 (5)	0.0312 (17)	
F5	0.1255 (16)	0.5093 (16)	0.5876 (9)	0.060 (3)	0.5
F5*	0.2022 (19)	0.5151 (17)	0.5939 (9)	0.065 (3)	0.5
F6	0.298 (3)	0.755 (3)	0.6206 (9)	0.040 (3)	0.5
F6*	0.303 (3)	0.762 (3)	0.5970 (11)	0.055 (5)	0.5
F7	0.1238 (17)	0.6497 (17)	0.5094 (8)	0.052 (3)	0.5
F7*	0.0761 (16)	0.5857 (18)	0.5254 (9)	0.058 (3)	0.5
F8	0.320 (2)	0.598 (2)	0.5211 (10)	0.057 (3)	0.5
F8*	0.298 (2)	0.606 (2)	0.4955 (8)	0.056 (4)	0.5
B2	0.2230 (11)	0.6249 (11)	0.5550 (6)	0.0387 (17)	

### [(1,2,5,6-η)-Cycloocta-1,5-diene](1-ethyl-4-isobutyl-1,2,4-triazol-5-ylidene)(triphenylphosphane)rhodium(I) tetrafluoridoborate Atomic displacement parameters (Å^2^)

**Table:**

	*U*^11^	*U*^22^	*U*^33^	*U*^12^	*U*^13^	*U*^23^
Rh1	0.0198 (2)	0.0243 (3)	0.0178 (2)	0.0156 (2)	0.00022 (18)	0.00819 (19)
P1	0.0199 (8)	0.0294 (9)	0.0187 (8)	0.0159 (7)	0.0008 (6)	0.0082 (7)
N1	0.024 (3)	0.041 (3)	0.025 (3)	0.022 (3)	−0.003 (2)	0.003 (2)
N2	0.029 (3)	0.036 (3)	0.032 (3)	0.024 (3)	0.000 (2)	0.006 (2)
N3	0.036 (3)	0.029 (3)	0.027 (3)	0.024 (3)	0.002 (2)	0.008 (2)
C1	0.027 (4)	0.031 (4)	0.021 (3)	0.017 (3)	0.001 (3)	0.009 (3)
C2	0.024 (3)	0.032 (3)	0.036 (4)	0.021 (3)	0.000 (3)	0.004 (3)
C3	0.016 (3)	0.036 (4)	0.045 (4)	0.009 (3)	0.003 (3)	0.005 (3)
C4	0.033 (4)	0.040 (4)	0.042 (4)	0.017 (3)	0.007 (3)	0.012 (3)
C5	0.023 (3)	0.037 (4)	0.040 (4)	0.018 (3)	0.002 (3)	0.014 (3)
C6	0.026 (3)	0.036 (4)	0.041 (4)	0.019 (3)	0.004 (3)	0.010 (3)
C7	0.029 (4)	0.036 (4)	0.061 (5)	0.017 (3)	0.009 (3)	0.025 (4)
C8	0.036 (4)	0.042 (4)	0.043 (4)	0.022 (4)	0.002 (3)	0.009 (3)
C9	0.026 (3)	0.037 (3)	0.021 (3)	0.020 (3)	0.006 (2)	0.015 (3)
C10	0.029 (3)	0.035 (3)	0.020 (3)	0.020 (3)	0.004 (2)	0.010 (3)
C11	0.028 (3)	0.038 (4)	0.034 (4)	0.018 (3)	0.007 (3)	0.016 (3)
C12	0.052 (5)	0.045 (4)	0.029 (4)	0.030 (4)	0.017 (3)	0.021 (3)
C13	0.043 (4)	0.055 (5)	0.022 (3)	0.033 (4)	0.006 (3)	0.017 (3)
C14	0.028 (3)	0.047 (4)	0.023 (3)	0.020 (3)	0.001 (3)	0.013 (3)
C15	0.021 (3)	0.036 (3)	0.025 (3)	0.020 (3)	−0.004 (2)	0.000 (3)
C16	0.027 (3)	0.052 (5)	0.028 (4)	0.021 (3)	0.001 (3)	−0.001 (3)
C17	0.032 (4)	0.068 (6)	0.039 (4)	0.030 (4)	0.001 (3)	−0.012 (4)
C18	0.037 (4)	0.050 (5)	0.049 (5)	0.028 (4)	−0.012 (4)	−0.016 (4)
C19	0.038 (4)	0.042 (4)	0.041 (4)	0.027 (4)	−0.014 (3)	−0.005 (4)
C20	0.028 (3)	0.031 (3)	0.027 (3)	0.020 (3)	−0.005 (3)	−0.002 (3)
C21	0.023 (3)	0.039 (4)	0.020 (3)	0.022 (3)	0.002 (2)	0.011 (3)
C22	0.025 (3)	0.040 (4)	0.023 (3)	0.020 (3)	0.001 (2)	0.014 (3)
C23	0.019 (3)	0.047 (4)	0.028 (3)	0.012 (3)	0.000 (3)	0.016 (3)
C24	0.028 (3)	0.059 (5)	0.033 (4)	0.032 (3)	0.005 (3)	0.016 (3)
C25	0.030 (3)	0.057 (5)	0.029 (3)	0.033 (3)	0.003 (3)	0.017 (3)
C26	0.028 (3)	0.038 (4)	0.021 (3)	0.021 (3)	−0.002 (2)	0.006 (3)
C27	0.0282 (16)	0.0274 (16)	0.0275 (16)	0.0205 (14)	−0.0028 (14)	0.0085 (14)
C28	0.0288 (16)	0.0275 (16)	0.0270 (16)	0.0208 (13)	−0.0035 (13)	0.0097 (13)
C29	0.0317 (16)	0.0304 (16)	0.0269 (16)	0.0168 (14)	−0.0035 (14)	0.0101 (14)
C30	0.0321 (16)	0.0310 (16)	0.0259 (16)	0.0168 (14)	−0.0036 (14)	0.0103 (14)
C31	0.0314 (16)	0.0301 (16)	0.0259 (16)	0.0178 (14)	−0.0033 (14)	0.0084 (14)
C32	0.0311 (16)	0.0294 (16)	0.0268 (16)	0.0173 (14)	−0.0033 (13)	0.0066 (14)
C33	0.0298 (16)	0.0290 (15)	0.0281 (16)	0.0176 (14)	−0.0035 (13)	0.0077 (14)
C34	0.0286 (16)	0.0283 (16)	0.0283 (16)	0.0187 (14)	−0.0026 (14)	0.0075 (14)
Rh1'	0.0168 (2)	0.0229 (3)	0.0164 (2)	0.0112 (2)	0.00022 (17)	0.00689 (18)
P1'	0.0186 (7)	0.0239 (8)	0.0170 (7)	0.0138 (6)	0.0007 (5)	0.0078 (6)
N1'	0.024 (3)	0.039 (3)	0.027 (3)	0.019 (2)	0.004 (2)	0.011 (3)
N2'	0.029 (3)	0.043 (3)	0.041 (3)	0.024 (3)	0.009 (3)	0.007 (3)
N3'	0.023 (3)	0.032 (3)	0.027 (3)	0.017 (2)	0.002 (2)	0.006 (2)
C1'	0.023 (3)	0.030 (3)	0.017 (3)	0.014 (3)	0.002 (3)	0.008 (3)
C2'	0.027 (3)	0.038 (4)	0.036 (4)	0.022 (3)	0.007 (3)	0.002 (3)
C3'	0.022 (3)	0.040 (4)	0.035 (4)	0.014 (3)	0.005 (3)	0.018 (3)
C4'	0.024 (3)	0.046 (4)	0.042 (4)	0.015 (3)	−0.002 (3)	0.009 (3)
C5'	0.030 (3)	0.029 (3)	0.027 (3)	0.020 (3)	0.000 (3)	0.006 (3)
C6'	0.033 (3)	0.032 (3)	0.030 (3)	0.021 (3)	0.001 (3)	0.006 (3)
C7'	0.032 (4)	0.036 (4)	0.041 (4)	0.014 (3)	−0.006 (3)	0.006 (3)
C8'	0.047 (5)	0.034 (4)	0.033 (4)	0.025 (4)	−0.005 (3)	0.004 (3)
C9'	0.023 (3)	0.025 (3)	0.019 (3)	0.014 (2)	−0.003 (2)	0.005 (2)
C10'	0.026 (3)	0.029 (3)	0.027 (3)	0.016 (3)	0.001 (2)	0.010 (3)
C11'	0.030 (3)	0.026 (3)	0.031 (3)	0.009 (3)	0.001 (3)	0.012 (3)
C12'	0.045 (4)	0.025 (3)	0.029 (3)	0.022 (3)	−0.009 (3)	0.004 (3)
C13'	0.031 (3)	0.032 (3)	0.027 (3)	0.023 (3)	−0.007 (3)	0.002 (3)
C14'	0.026 (3)	0.029 (3)	0.020 (3)	0.015 (3)	0.000 (2)	0.006 (2)
C15'	0.031 (3)	0.029 (3)	0.020 (3)	0.021 (3)	0.007 (2)	0.015 (2)
C16'	0.027 (3)	0.033 (3)	0.029 (3)	0.016 (3)	0.005 (3)	0.017 (3)
C17'	0.026 (3)	0.044 (4)	0.042 (4)	0.020 (3)	0.009 (3)	0.022 (3)
C18'	0.039 (4)	0.055 (5)	0.043 (4)	0.035 (4)	0.021 (3)	0.029 (4)
C19'	0.044 (4)	0.039 (4)	0.030 (3)	0.031 (3)	0.013 (3)	0.013 (3)
C20'	0.029 (3)	0.033 (3)	0.024 (3)	0.019 (3)	0.003 (3)	0.013 (3)
C21'	0.013 (2)	0.024 (3)	0.019 (3)	0.008 (2)	−0.001 (2)	0.004 (2)
C22'	0.023 (3)	0.031 (3)	0.024 (3)	0.011 (3)	−0.001 (2)	0.010 (3)
C23'	0.031 (4)	0.026 (3)	0.037 (4)	0.008 (3)	0.003 (3)	0.012 (3)
C24'	0.025 (3)	0.036 (4)	0.028 (3)	0.011 (3)	−0.002 (3)	0.003 (3)
C25'	0.026 (3)	0.043 (4)	0.023 (3)	0.022 (3)	−0.003 (2)	0.005 (3)
C26'	0.026 (3)	0.033 (3)	0.023 (3)	0.021 (3)	0.000 (2)	0.007 (3)
C27'	0.018 (3)	0.030 (3)	0.023 (3)	0.016 (3)	0.002 (2)	0.013 (3)
C28'	0.030 (4)	0.027 (4)	0.022 (3)	0.020 (3)	0.000 (3)	0.008 (3)
C29'	0.027 (3)	0.020 (3)	0.040 (4)	0.012 (3)	−0.003 (3)	0.013 (3)
C30'	0.027 (3)	0.027 (3)	0.027 (3)	0.009 (3)	0.000 (3)	0.012 (3)
C31'	0.022 (3)	0.037 (3)	0.018 (3)	0.013 (3)	0.002 (2)	0.015 (3)
C32'	0.026 (4)	0.037 (4)	0.014 (3)	0.015 (3)	0.002 (2)	0.006 (3)
C33'	0.021 (3)	0.027 (3)	0.020 (3)	0.010 (3)	−0.001 (2)	0.009 (2)
C34'	0.020 (3)	0.031 (3)	0.025 (3)	0.012 (3)	−0.002 (2)	0.013 (3)
F1	0.043 (3)	0.042 (3)	0.085 (4)	0.026 (2)	0.030 (3)	0.029 (3)
F2	0.071 (4)	0.043 (3)	0.076 (4)	0.042 (3)	0.037 (3)	0.031 (3)
F3	0.085 (4)	0.096 (5)	0.051 (3)	0.076 (4)	−0.032 (3)	−0.020 (3)
F4	0.046 (3)	0.042 (2)	0.034 (2)	0.031 (2)	0.0011 (19)	0.0067 (19)
B1	0.034 (4)	0.037 (4)	0.032 (4)	0.024 (4)	−0.002 (3)	0.014 (3)
F5	0.053 (6)	0.047 (5)	0.070 (6)	0.012 (5)	0.012 (6)	0.029 (4)
F5*	0.076 (7)	0.057 (5)	0.069 (6)	0.034 (6)	0.006 (6)	0.032 (5)
F6	0.036 (5)	0.039 (5)	0.036 (7)	0.015 (4)	−0.012 (6)	0.006 (6)
F6*	0.042 (5)	0.040 (6)	0.068 (10)	0.012 (5)	0.005 (9)	0.003 (9)
F7	0.049 (7)	0.069 (7)	0.050 (6)	0.039 (6)	−0.010 (5)	0.020 (5)
F7*	0.037 (6)	0.072 (7)	0.067 (7)	0.033 (5)	−0.003 (5)	0.001 (6)
F8	0.051 (6)	0.067 (5)	0.078 (8)	0.048 (4)	0.010 (6)	0.019 (6)
F8*	0.044 (6)	0.082 (7)	0.052 (7)	0.032 (5)	0.020 (6)	0.032 (6)
B2	0.033 (3)	0.045 (4)	0.050 (4)	0.027 (3)	0.006 (3)	0.019 (3)

### [(1,2,5,6-η)-Cycloocta-1,5-diene](1-ethyl-4-isobutyl-1,2,4-triazol-5-ylidene)(triphenylphosphane)rhodium(I) tetrafluoridoborate Geometric parameters (Å, º)

**Table:**

Rh1—P1	2.3211 (18)	P1'—C9'	1.828 (7)
Rh1—C1	2.030 (8)	P1'—C15'	1.824 (7)
Rh1—C27	2.193 (8)	P1'—C21'	1.822 (6)
Rh1—C28	2.204 (6)	N1'—N2'	1.377 (9)
Rh1—C31	2.210 (7)	N1'—C1'	1.328 (9)
Rh1—C32	2.232 (7)	N1'—C3'	1.457 (10)
P1—C9	1.831 (7)	N2'—C2'	1.282 (10)
P1—C15	1.842 (7)	N3'—C1'	1.362 (10)
P1—C21	1.842 (7)	N3'—C2'	1.366 (9)
N1—N2	1.359 (8)	N3'—C5'	1.467 (9)
N1—C1	1.353 (10)	C2'—H2'	0.9500
N1—C3	1.471 (10)	C3'—H3'A	0.9900
N2—C2	1.271 (10)	C3'—H3'B	0.9900
N3—C1	1.363 (10)	C3'—C4'	1.528 (10)
N3—C2	1.384 (9)	C4'—H4'A	0.9800
N3—C5	1.444 (10)	C4'—H4'B	0.9800
C2—H2	0.9500	C4'—H4'C	0.9800
C3—H3A	0.9900	C5'—H5'A	0.9900
C3—H3B	0.9900	C5'—H5'B	0.9900
C3—C4	1.513 (11)	C5'—C6'	1.533 (9)
C4—H4A	0.9800	C6'—H6'	1.0000
C4—H4B	0.9800	C6'—C7'	1.512 (11)
C4—H4C	0.9800	C6'—C8'	1.526 (11)
C5—H5A	0.9900	C7'—H7'A	0.9800
C5—H5B	0.9900	C7'—H7'B	0.9800
C5—C6	1.494 (11)	C7'—H7'C	0.9800
C6—H6	1.0000	C8'—H8'A	0.9800
C6—C7	1.529 (11)	C8'—H8'B	0.9800
C6—C8	1.525 (11)	C8'—H8'C	0.9800
C7—H7A	0.9800	C9'—C10'	1.395 (9)
C7—H7B	0.9800	C9'—C14'	1.405 (9)
C7—H7C	0.9800	C10'—H10'	0.9500
C8—H8A	0.9800	C10'—C11'	1.397 (10)
C8—H8B	0.9800	C11'—H11'	0.9500
C8—H8C	0.9800	C11'—C12'	1.401 (11)
C9—C10	1.391 (10)	C12'—H12'	0.9500
C9—C14	1.413 (9)	C12'—C13'	1.373 (11)
C10—H10	0.9500	C13'—H13'	0.9500
C10—C11	1.379 (10)	C13'—C14'	1.393 (10)
C11—H11	0.9500	C14'—H14'	0.9500
C11—C12	1.402 (10)	C15'—C16'	1.408 (10)
C12—H12	0.9500	C15'—C20'	1.391 (10)
C12—C13	1.394 (12)	C16'—H16'	0.9500
C13—H13	0.9500	C16'—C17'	1.395 (10)
C13—C14	1.363 (11)	C17'—H17'	0.9500
C14—H14	0.9500	C17'—C18'	1.381 (12)
C15—C16	1.402 (10)	C18'—H18'	0.9500
C15—C20	1.384 (11)	C18'—C19'	1.386 (12)
C16—H16	0.9500	C19'—H19'	0.9500
C16—C17	1.371 (12)	C19'—C20'	1.395 (10)
C17—H17	0.9500	C20'—H20'	0.9500
C17—C18	1.392 (15)	C21'—C22'	1.391 (9)
C18—H18	0.9500	C21'—C26'	1.411 (8)
C18—C19	1.363 (13)	C22'—H22'	0.9500
C19—H19	0.9500	C22'—C23'	1.386 (10)
C19—C20	1.406 (11)	C23'—H23'	0.9500
C20—H20	0.9500	C23'—C24'	1.390 (10)
C21—C22	1.385 (10)	C24'—H24'	0.9500
C21—C26	1.401 (10)	C24'—C25'	1.365 (11)
C22—H22	0.9500	C25'—H25'	0.9500
C22—C23	1.385 (9)	C25'—C26'	1.402 (9)
C23—H23	0.9500	C26'—H26'	0.9500
C23—C24	1.372 (12)	C27'—H27'	1.0000
C24—H24	0.9500	C27'—C28'	1.386 (10)
C24—C25	1.394 (12)	C27'—C34'	1.518 (9)
C25—H25	0.9500	C28'—H28'	1.0000
C25—C26	1.396 (9)	C28'—C29'	1.501 (10)
C26—H26	0.9500	C29'—H29C	0.9900
C27—H27	1.0000	C29'—H29D	0.9900
C27—C28	1.376 (11)	C29'—C30'	1.534 (10)
C27—C34	1.505 (11)	C30'—H30C	0.9900
C28—H28	1.0000	C30'—H30D	0.9900
C28—C29	1.524 (9)	C30'—C31'	1.513 (10)
C29—H29A	0.9900	C31'—H31'	1.0000
C29—H29B	0.9900	C31'—C32'	1.383 (10)
C29—C30	1.518 (10)	C32'—H32'	1.0000
C30—H30A	0.9900	C32'—C33'	1.486 (10)
C30—H30B	0.9900	C33'—H33C	0.9900
C30—C31	1.535 (10)	C33'—H33D	0.9900
C31—H31	1.0000	C33'—C34'	1.534 (10)
C31—C32	1.374 (11)	C34'—H34C	0.9900
C32—H32	1.0000	C34'—H34D	0.9900
C32—C33	1.535 (10)	F1—B1	1.407 (11)
C33—H33A	0.9900	F2—B1	1.376 (10)
C33—H33B	0.9900	F3—B1	1.402 (10)
C33—C34	1.542 (10)	F4—B1	1.372 (11)
C34—H34A	0.9900	F5—B2	1.386 (16)
C34—H34B	0.9900	F5*—B2	1.374 (17)
Rh1'—P1'	2.3260 (15)	F6—B2	1.48 (2)
Rh1'—C1'	2.043 (8)	F6*—B2	1.31 (3)
Rh1'—C27'	2.229 (6)	F7—B2	1.394 (15)
Rh1'—C28'	2.211 (7)	F7*—B2	1.367 (16)
Rh1'—C31'	2.216 (6)	F8—B2	1.30 (2)
Rh1'—C32'	2.216 (7)	F8*—B2	1.40 (2)
			
C1—Rh1—P1	89.6 (2)	C28'—Rh1'—C32'	95.5 (3)
C1—Rh1—C27	168.5 (3)	C31'—Rh1'—P1'	157.09 (18)
C1—Rh1—C28	154.6 (3)	C31'—Rh1'—C27'	86.1 (2)
C1—Rh1—C31	85.6 (3)	C32'—Rh1'—P1'	166.5 (2)
C1—Rh1—C32	95.0 (3)	C32'—Rh1'—C27'	79.8 (3)
C27—Rh1—P1	90.3 (2)	C32'—Rh1'—C31'	36.4 (3)
C27—Rh1—C28	36.5 (3)	C9'—P1'—Rh1'	118.0 (2)
C27—Rh1—C31	96.6 (3)	C15'—P1'—Rh1'	114.5 (2)
C27—Rh1—C32	80.6 (3)	C15'—P1'—C9'	105.8 (3)
C28—Rh1—P1	98.75 (18)	C21'—P1'—Rh1'	109.4 (2)
C28—Rh1—C31	81.3 (3)	C21'—P1'—C9'	103.9 (3)
C28—Rh1—C32	87.1 (3)	C21'—P1'—C15'	103.7 (3)
C31—Rh1—P1	167.8 (2)	N2'—N1'—C3'	117.9 (6)
C31—Rh1—C32	36.0 (3)	C1'—N1'—N2'	113.4 (6)
C32—Rh1—P1	156.0 (2)	C1'—N1'—C3'	128.7 (6)
C9—P1—Rh1	109.1 (2)	C2'—N2'—N1'	103.1 (6)
C9—P1—C15	104.3 (3)	C1'—N3'—C2'	107.2 (6)
C9—P1—C21	103.9 (3)	C1'—N3'—C5'	125.1 (6)
C15—P1—Rh1	116.3 (2)	C2'—N3'—C5'	127.5 (6)
C15—P1—C21	105.9 (3)	N1'—C1'—Rh1'	130.2 (6)
C21—P1—Rh1	116.0 (2)	N1'—C1'—N3'	103.8 (6)
N2—N1—C3	119.7 (6)	N3'—C1'—Rh1'	126.0 (5)
C1—N1—N2	114.8 (6)	N2'—C2'—N3'	112.4 (7)
C1—N1—C3	125.3 (6)	N2'—C2'—H2'	123.8
C2—N2—N1	102.8 (6)	N3'—C2'—H2'	123.8
C1—N3—C2	106.9 (6)	N1'—C3'—H3'A	109.2
C1—N3—C5	125.0 (6)	N1'—C3'—H3'B	109.2
C2—N3—C5	127.7 (6)	N1'—C3'—C4'	112.1 (6)
N1—C1—Rh1	124.4 (6)	H3'A—C3'—H3'B	107.9
N1—C1—N3	102.4 (7)	C4'—C3'—H3'A	109.2
N3—C1—Rh1	133.2 (6)	C4'—C3'—H3'B	109.2
N2—C2—N3	113.1 (6)	C3'—C4'—H4'A	109.5
N2—C2—H2	123.4	C3'—C4'—H4'B	109.5
N3—C2—H2	123.4	C3'—C4'—H4'C	109.5
N1—C3—H3A	109.2	H4'A—C4'—H4'B	109.5
N1—C3—H3B	109.2	H4'A—C4'—H4'C	109.5
N1—C3—C4	111.9 (6)	H4'B—C4'—H4'C	109.5
H3A—C3—H3B	107.9	N3'—C5'—H5'A	108.9
C4—C3—H3A	109.2	N3'—C5'—H5'B	108.9
C4—C3—H3B	109.2	N3'—C5'—C6'	113.5 (6)
C3—C4—H4A	109.5	H5'A—C5'—H5'B	107.7
C3—C4—H4B	109.5	C6'—C5'—H5'A	108.9
C3—C4—H4C	109.5	C6'—C5'—H5'B	108.9
H4A—C4—H4B	109.5	C5'—C6'—H6'	108.1
H4A—C4—H4C	109.5	C7'—C6'—C5'	109.1 (6)
H4B—C4—H4C	109.5	C7'—C6'—H6'	108.1
N3—C5—H5A	108.8	C7'—C6'—C8'	111.6 (6)
N3—C5—H5B	108.8	C8'—C6'—C5'	111.7 (6)
N3—C5—C6	113.7 (6)	C8'—C6'—H6'	108.1
H5A—C5—H5B	107.7	C6'—C7'—H7'A	109.5
C6—C5—H5A	108.8	C6'—C7'—H7'B	109.5
C6—C5—H5B	108.8	C6'—C7'—H7'C	109.5
C5—C6—H6	108.7	H7'A—C7'—H7'B	109.5
C5—C6—C7	109.5 (7)	H7'A—C7'—H7'C	109.5
C5—C6—C8	110.0 (6)	H7'B—C7'—H7'C	109.5
C7—C6—H6	108.7	C6'—C8'—H8'A	109.5
C8—C6—H6	108.7	C6'—C8'—H8'B	109.5
C8—C6—C7	111.3 (6)	C6'—C8'—H8'C	109.5
C6—C7—H7A	109.5	H8'A—C8'—H8'B	109.5
C6—C7—H7B	109.5	H8'A—C8'—H8'C	109.5
C6—C7—H7C	109.5	H8'B—C8'—H8'C	109.5
H7A—C7—H7B	109.5	C10'—C9'—P1'	123.0 (5)
H7A—C7—H7C	109.5	C10'—C9'—C14'	118.1 (6)
H7B—C7—H7C	109.5	C14'—C9'—P1'	118.7 (5)
C6—C8—H8A	109.5	C9'—C10'—H10'	119.7
C6—C8—H8B	109.5	C9'—C10'—C11'	120.7 (6)
C6—C8—H8C	109.5	C11'—C10'—H10'	119.7
H8A—C8—H8B	109.5	C10'—C11'—H11'	120.0
H8A—C8—H8C	109.5	C10'—C11'—C12'	120.0 (6)
H8B—C8—H8C	109.5	C12'—C11'—H11'	120.0
C10—C9—P1	119.0 (5)	C11'—C12'—H12'	120.1
C10—C9—C14	118.6 (6)	C13'—C12'—C11'	119.8 (6)
C14—C9—P1	122.5 (5)	C13'—C12'—H12'	120.1
C9—C10—H10	119.4	C12'—C13'—H13'	119.9
C11—C10—C9	121.2 (6)	C12'—C13'—C14'	120.2 (7)
C11—C10—H10	119.4	C14'—C13'—H13'	119.9
C10—C11—H11	120.2	C9'—C14'—H14'	119.4
C10—C11—C12	119.6 (7)	C13'—C14'—C9'	121.2 (6)
C12—C11—H11	120.2	C13'—C14'—H14'	119.4
C11—C12—H12	120.3	C16'—C15'—P1'	118.5 (5)
C13—C12—C11	119.4 (7)	C20'—C15'—P1'	122.4 (5)
C13—C12—H12	120.3	C20'—C15'—C16'	118.7 (6)
C12—C13—H13	119.6	C15'—C16'—H16'	120.2
C14—C13—C12	120.8 (7)	C17'—C16'—C15'	119.5 (7)
C14—C13—H13	119.6	C17'—C16'—H16'	120.2
C9—C14—H14	119.8	C16'—C17'—H17'	119.5
C13—C14—C9	120.4 (7)	C18'—C17'—C16'	121.0 (7)
C13—C14—H14	119.8	C18'—C17'—H17'	119.5
C16—C15—P1	121.6 (6)	C17'—C18'—H18'	120.1
C20—C15—P1	119.0 (5)	C17'—C18'—C19'	119.9 (7)
C20—C15—C16	119.2 (7)	C19'—C18'—H18'	120.1
C15—C16—H16	120.1	C18'—C19'—H19'	120.2
C17—C16—C15	119.8 (8)	C18'—C19'—C20'	119.6 (7)
C17—C16—H16	120.1	C20'—C19'—H19'	120.2
C16—C17—H17	119.2	C15'—C20'—C19'	121.2 (6)
C16—C17—C18	121.6 (8)	C15'—C20'—H20'	119.4
C18—C17—H17	119.2	C19'—C20'—H20'	119.4
C17—C18—H18	120.7	C22'—C21'—P1'	119.8 (5)
C19—C18—C17	118.6 (8)	C22'—C21'—C26'	118.7 (6)
C19—C18—H18	120.7	C26'—C21'—P1'	121.6 (5)
C18—C19—H19	119.4	C21'—C22'—H22'	119.5
C18—C19—C20	121.2 (9)	C23'—C22'—C21'	121.1 (6)
C20—C19—H19	119.4	C23'—C22'—H22'	119.5
C15—C20—C19	119.6 (7)	C22'—C23'—H23'	120.1
C15—C20—H20	120.2	C22'—C23'—C24'	119.7 (7)
C19—C20—H20	120.2	C24'—C23'—H23'	120.1
C22—C21—P1	123.1 (5)	C23'—C24'—H24'	119.8
C22—C21—C26	118.9 (6)	C25'—C24'—C23'	120.4 (6)
C26—C21—P1	118.0 (5)	C25'—C24'—H24'	119.8
C21—C22—H22	119.8	C24'—C25'—H25'	119.6
C21—C22—C23	120.4 (7)	C24'—C25'—C26'	120.7 (6)
C23—C22—H22	119.8	C26'—C25'—H25'	119.6
C22—C23—H23	119.5	C21'—C26'—H26'	120.3
C24—C23—C22	120.9 (7)	C25'—C26'—C21'	119.4 (6)
C24—C23—H23	119.5	C25'—C26'—H26'	120.3
C23—C24—H24	120.1	Rh1'—C27'—H27'	113.8
C23—C24—C25	119.8 (6)	C28'—C27'—Rh1'	71.1 (4)
C25—C24—H24	120.1	C28'—C27'—H27'	113.8
C24—C25—H25	120.3	C28'—C27'—C34'	125.3 (6)
C24—C25—C26	119.5 (7)	C34'—C27'—Rh1'	111.3 (4)
C26—C25—H25	120.3	C34'—C27'—H27'	113.8
C21—C26—H26	119.8	Rh1'—C28'—H28'	114.0
C25—C26—C21	120.5 (7)	C27'—C28'—Rh1'	72.5 (4)
C25—C26—H26	119.8	C27'—C28'—H28'	114.0
Rh1—C27—H27	113.8	C27'—C28'—C29'	126.0 (6)
C28—C27—Rh1	72.2 (4)	C29'—C28'—Rh1'	108.3 (5)
C28—C27—H27	113.8	C29'—C28'—H28'	114.0
C28—C27—C34	125.9 (7)	C28'—C29'—H29C	108.9
C34—C27—Rh1	109.4 (5)	C28'—C29'—H29D	108.9
C34—C27—H27	113.8	C28'—C29'—C30'	113.5 (6)
Rh1—C28—H28	113.6	H29C—C29'—H29D	107.7
C27—C28—Rh1	71.3 (4)	C30'—C29'—H29C	108.9
C27—C28—H28	113.6	C30'—C29'—H29D	108.9
C27—C28—C29	125.7 (6)	C29'—C30'—H30C	109.1
C29—C28—Rh1	111.5 (5)	C29'—C30'—H30D	109.1
C29—C28—H28	113.6	H30C—C30'—H30D	107.9
C28—C29—H29A	108.9	C31'—C30'—C29'	112.4 (5)
C28—C29—H29B	108.9	C31'—C30'—H30C	109.1
H29A—C29—H29B	107.7	C31'—C30'—H30D	109.1
C30—C29—C28	113.4 (6)	Rh1'—C31'—H31'	113.2
C30—C29—H29A	108.9	C30'—C31'—Rh1'	112.6 (4)
C30—C29—H29B	108.9	C30'—C31'—H31'	113.2
C29—C30—H30A	108.8	C32'—C31'—Rh1'	71.8 (4)
C29—C30—H30B	108.8	C32'—C31'—C30'	125.6 (6)
C29—C30—C31	113.9 (6)	C32'—C31'—H31'	113.2
H30A—C30—H30B	107.7	Rh1'—C32'—H32'	113.8
C31—C30—H30A	108.8	C31'—C32'—Rh1'	71.9 (4)
C31—C30—H30B	108.8	C31'—C32'—H32'	113.8
Rh1—C31—H31	114.6	C31'—C32'—C33'	127.1 (7)
C30—C31—Rh1	106.5 (5)	C33'—C32'—Rh1'	107.9 (5)
C30—C31—H31	114.6	C33'—C32'—H32'	113.8
C32—C31—Rh1	72.9 (4)	C32'—C33'—H33C	109.0
C32—C31—C30	125.2 (7)	C32'—C33'—H33D	109.0
C32—C31—H31	114.6	C32'—C33'—C34'	113.0 (6)
Rh1—C32—H32	113.9	H33C—C33'—H33D	107.8
C31—C32—Rh1	71.1 (4)	C34'—C33'—H33C	109.0
C31—C32—H32	113.9	C34'—C33'—H33D	109.0
C31—C32—C33	125.2 (6)	C27'—C34'—C33'	112.8 (5)
C33—C32—Rh1	110.9 (5)	C27'—C34'—H34C	109.0
C33—C32—H32	113.9	C27'—C34'—H34D	109.0
C32—C33—H33A	108.9	C33'—C34'—H34C	109.0
C32—C33—H33B	108.9	C33'—C34'—H34D	109.0
C32—C33—C34	113.4 (5)	H34C—C34'—H34D	107.8
H33A—C33—H33B	107.7	F2—B1—F1	107.8 (7)
C34—C33—H33A	108.9	F2—B1—F3	109.5 (8)
C34—C33—H33B	108.9	F3—B1—F1	110.0 (8)
C27—C34—C33	112.7 (6)	F4—B1—F1	108.0 (7)
C27—C34—H34A	109.0	F4—B1—F2	110.9 (7)
C27—C34—H34B	109.0	F4—B1—F3	110.5 (6)
C33—C34—H34A	109.0	F5—B2—F6	102.2 (12)
C33—C34—H34B	109.0	F5—B2—F7	104.3 (11)
H34A—C34—H34B	107.8	F5*—B2—F8*	106.1 (12)
C1'—Rh1'—P1'	89.8 (2)	F6*—B2—F5*	113.4 (14)
C1'—Rh1'—C27'	159.2 (3)	F6*—B2—F7*	113.8 (17)
C1'—Rh1'—C28'	163.4 (3)	F6*—B2—F8*	110.8 (16)
C1'—Rh1'—C31'	93.4 (3)	F7—B2—F6	106.1 (14)
C1'—Rh1'—C32'	87.5 (3)	F7*—B2—F5*	103.7 (12)
C27'—Rh1'—P1'	98.72 (16)	F7*—B2—F8*	108.5 (11)
C28'—Rh1'—P1'	90.87 (19)	F8—B2—F5	113.8 (13)
C28'—Rh1'—C27'	36.4 (3)	F8—B2—F6	113.7 (15)
C28'—Rh1'—C31'	79.7 (3)	F8—B2—F7	115.5 (12)
			
Rh1—P1—C9—C10	24.0 (6)	Rh1'—P1'—C9'—C10'	109.3 (5)
Rh1—P1—C9—C14	−154.5 (6)	Rh1'—P1'—C9'—C14'	−66.0 (5)
Rh1—P1—C15—C16	−116.9 (5)	Rh1'—P1'—C15'—C16'	−62.2 (5)
Rh1—P1—C15—C20	59.3 (6)	Rh1'—P1'—C15'—C20'	110.6 (5)
Rh1—P1—C21—C22	−105.0 (5)	Rh1'—P1'—C21'—C22'	−28.4 (6)
Rh1—P1—C21—C26	71.7 (6)	Rh1'—P1'—C21'—C26'	150.9 (5)
Rh1—C27—C28—C29	−103.6 (7)	Rh1'—C27'—C28'—C29'	−100.2 (7)
Rh1—C27—C34—C33	39.8 (7)	Rh1'—C27'—C34'—C33'	−13.2 (7)
Rh1—C28—C29—C30	11.8 (7)	Rh1'—C28'—C29'—C30'	−40.7 (7)
Rh1—C31—C32—C33	−102.7 (7)	Rh1'—C31'—C32'—C33'	−99.1 (7)
Rh1—C32—C33—C34	13.4 (7)	Rh1'—C32'—C33'—C34'	−42.9 (7)
P1—C9—C10—C11	−179.0 (6)	P1'—C9'—C10'—C11'	−174.7 (5)
P1—C9—C14—C13	178.3 (6)	P1'—C9'—C14'—C13'	173.9 (5)
P1—C15—C16—C17	174.1 (6)	P1'—C15'—C16'—C17'	173.2 (5)
P1—C15—C20—C19	−173.8 (5)	P1'—C15'—C20'—C19'	−173.2 (5)
P1—C21—C22—C23	174.9 (5)	P1'—C21'—C22'—C23'	−178.5 (6)
P1—C21—C26—C25	−174.3 (5)	P1'—C21'—C26'—C25'	179.7 (5)
N1—N2—C2—N3	0.2 (8)	N1'—N2'—C2'—N3'	−0.5 (9)
N2—N1—C1—Rh1	178.5 (5)	N2'—N1'—C1'—Rh1'	179.5 (5)
N2—N1—C1—N3	0.6 (8)	N2'—N1'—C1'—N3'	0.8 (8)
N2—N1—C3—C4	−57.3 (8)	N2'—N1'—C3'—C4'	58.1 (9)
N3—C5—C6—C7	−170.1 (6)	N3'—C5'—C6'—C7'	−171.6 (6)
N3—C5—C6—C8	67.3 (8)	N3'—C5'—C6'—C8'	64.6 (8)
C1—N1—N2—C2	−0.5 (8)	C1'—N1'—N2'—C2'	−0.2 (8)
C1—N1—C3—C4	128.9 (7)	C1'—N1'—C3'—C4'	−123.0 (8)
C1—N3—C2—N2	0.2 (8)	C1'—N3'—C2'—N2'	1.0 (9)
C1—N3—C5—C6	−142.1 (7)	C1'—N3'—C5'—C6'	−134.7 (7)
C2—N3—C1—Rh1	−178.1 (6)	C2'—N3'—C1'—Rh1'	−179.8 (5)
C2—N3—C1—N1	−0.4 (7)	C2'—N3'—C1'—N1'	−1.1 (8)
C2—N3—C5—C6	46.2 (10)	C2'—N3'—C5'—C6'	50.7 (9)
C3—N1—N2—C2	−174.9 (6)	C3'—N1'—N2'—C2'	178.9 (6)
C3—N1—C1—Rh1	−7.4 (10)	C3'—N1'—C1'—Rh1'	0.5 (11)
C3—N1—C1—N3	174.6 (6)	C3'—N1'—C1'—N3'	−178.2 (6)
C5—N3—C1—Rh1	8.7 (11)	C5'—N3'—C1'—Rh1'	4.7 (10)
C5—N3—C1—N1	−173.6 (6)	C5'—N3'—C1'—N1'	−176.5 (6)
C5—N3—C2—N2	173.1 (7)	C5'—N3'—C2'—N2'	176.4 (7)
C9—P1—C15—C16	3.2 (6)	C9'—P1'—C15'—C16'	69.6 (5)
C9—P1—C15—C20	179.4 (5)	C9'—P1'—C15'—C20'	−117.7 (5)
C9—P1—C21—C22	135.3 (6)	C9'—P1'—C21'—C22'	−155.3 (5)
C9—P1—C21—C26	−48.0 (6)	C9'—P1'—C21'—C26'	24.0 (6)
C9—C10—C11—C12	2.1 (11)	C9'—C10'—C11'—C12'	0.9 (10)
C10—C9—C14—C13	−0.2 (11)	C10'—C9'—C14'—C13'	−1.7 (9)
C10—C11—C12—C13	−3.0 (12)	C10'—C11'—C12'—C13'	−1.5 (10)
C11—C12—C13—C14	2.3 (12)	C11'—C12'—C13'—C14'	0.5 (10)
C12—C13—C14—C9	−0.7 (12)	C12'—C13'—C14'—C9'	1.2 (10)
C14—C9—C10—C11	−0.5 (10)	C14'—C9'—C10'—C11'	0.7 (9)
C15—P1—C9—C10	−100.9 (6)	C15'—P1'—C9'—C10'	−20.4 (6)
C15—P1—C9—C14	80.6 (6)	C15'—P1'—C9'—C14'	164.3 (5)
C15—P1—C21—C22	25.7 (6)	C15'—P1'—C21'—C22'	94.3 (6)
C15—P1—C21—C26	−157.6 (5)	C15'—P1'—C21'—C26'	−86.5 (6)
C15—C16—C17—C18	0.4 (12)	C15'—C16'—C17'—C18'	−0.1 (11)
C16—C15—C20—C19	2.6 (10)	C16'—C15'—C20'—C19'	−0.5 (9)
C16—C17—C18—C19	0.9 (12)	C16'—C17'—C18'—C19'	0.4 (11)
C17—C18—C19—C20	−0.5 (12)	C17'—C18'—C19'—C20'	−0.7 (11)
C18—C19—C20—C15	−1.3 (11)	C18'—C19'—C20'—C15'	0.8 (10)
C20—C15—C16—C17	−2.2 (10)	C20'—C15'—C16'—C17'	0.1 (9)
C21—P1—C9—C10	148.3 (6)	C21'—P1'—C9'—C10'	−129.3 (5)
C21—P1—C9—C14	−30.2 (7)	C21'—P1'—C9'—C14'	55.3 (5)
C21—P1—C15—C16	112.5 (6)	C21'—P1'—C15'—C16'	178.6 (5)
C21—P1—C15—C20	−71.2 (6)	C21'—P1'—C15'—C20'	−8.6 (6)
C21—C22—C23—C24	0.5 (10)	C21'—C22'—C23'—C24'	−1.9 (12)
C22—C21—C26—C25	2.5 (10)	C22'—C21'—C26'—C25'	−1.0 (9)
C22—C23—C24—C25	0.1 (11)	C22'—C23'—C24'—C25'	0.4 (12)
C23—C24—C25—C26	0.6 (11)	C23'—C24'—C25'—C26'	0.7 (11)
C24—C25—C26—C21	−2.0 (10)	C24'—C25'—C26'—C21'	−0.4 (10)
C26—C21—C22—C23	−1.8 (10)	C26'—C21'—C22'—C23'	2.2 (10)
C27—C28—C29—C30	93.7 (9)	C27'—C28'—C29'—C30'	40.6 (10)
C28—C27—C34—C33	−41.8 (10)	C28'—C27'—C34'—C33'	−94.6 (8)
C28—C29—C30—C31	−35.6 (8)	C28'—C29'—C30'—C31'	35.4 (8)
C29—C30—C31—Rh1	40.3 (7)	C29'—C30'—C31'—Rh1'	−11.8 (7)
C29—C30—C31—C32	−40.1 (10)	C29'—C30'—C31'—C32'	−95.0 (8)
C30—C31—C32—Rh1	98.4 (7)	C30'—C31'—C32'—Rh1'	105.2 (6)
C30—C31—C32—C33	−4.3 (11)	C30'—C31'—C32'—C33'	6.1 (11)
C31—C32—C33—C34	94.5 (8)	C31'—C32'—C33'—C34'	37.6 (9)
C32—C33—C34—C27	−35.7 (8)	C32'—C33'—C34'—C27'	38.1 (8)
C34—C27—C28—Rh1	101.4 (8)	C34'—C27'—C28'—Rh1'	103.2 (6)
C34—C27—C28—C29	−2.1 (12)	C34'—C27'—C28'—C29'	3.0 (11)

### [(1,2,5,6-η)-Cycloocta-1,5-diene](1-ethyl-4-isobutyl-1,2,4-triazol-5-ylidene)(triphenylphosphane)rhodium(I) tetrafluoridoborate Hydrogen-bond geometry (Å, º)

**Table:**

*D*—H···*A*	*D*—H	H···*A*	*D*···*A*	*D*—H···*A*
C2—H2···F3^i^	0.95	2.36	3.177 (8)	144
C8—H8*C*···F1	0.98	2.50	3.404 (10)	153
C13—H13···F5*	0.95	2.52	3.396 (17)	153
C20—H20···F2^ii^	0.95	2.34	3.291 (9)	178
C16′—H16′···F7*	0.95	2.40	3.351 (17)	177
C29′—H29*D*···F8^ii^	0.99	2.51	3.37 (2)	146

Symmetry codes: (i) *x*−1, *y*, *z*; (ii) *x*−1, *y*−1, *z*.
